# Many Ways Out: Beyond the Two-State Model of Protein Unfolding

**DOI:** 10.1021/acsomega.6c02203

**Published:** 2026-06-04

**Authors:** Annalisa Pastore, Piero Andrea Temussi

**Affiliations:** † Department of Clinical and Basic Neurosciences, King’s College London, SW59RT London, U.K.; ‡ Department of Engineering, King’s College London, The Strand, WC2R 2LS London, U.K.; § Elettra Sincrotrone Trieste, Area Science Park, Basovizza, Trieste 34149, Italy; ∥ Department of Chemistry, Universita’ Federico II, 80100 Napoli, Italy

## Abstract

The conformational transition related to protein unfolding is often considered as the transition between *the* folded conformation and *one* unfolded form. However, there are many aspects that hint at a much richer and more complex unfolded state made of a conformational ensemble not only for the unfolded species but also during the unfolding process. Proteins often sample intermediate conformations that may retain elements of secondary structure, preserve part of the hydrophobic core, or display localized unfolding restricted to specific regions. During the unfolding pathway, there are often minor transitions involving local secondary-structure regions outside the hydrophobic core. Many researchers have also hypothesized the existence of one or more intermediates, albeit usually invisible because they are low populated. In the present Mini-Review, we re-examine critically crucial aspects of this fascinating problem starting from our own experience in protein stability and unfolding and revise the limitations and implications of the two-state model.

## Graphical abstract

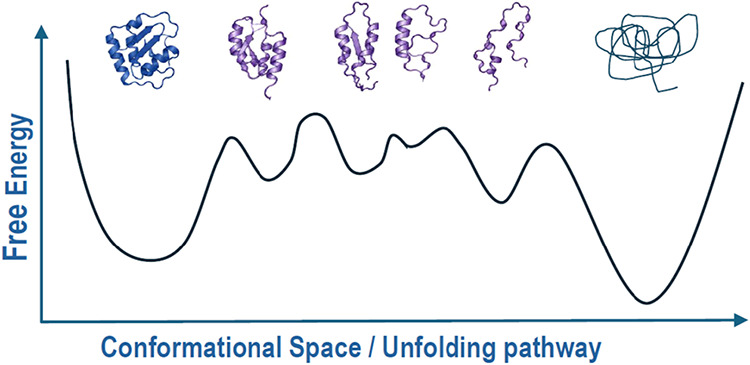

## Introduction

Most people, when talking about conformational transitions related to protein unfolding, think immediately of the transition between *the* folded conformation and *an* unfolded form. This restricted view stems from the “two-state” model that applies to the unfolding process of many single domain proteins. However, there are many aspects that hint at a rich and complex conformational ensemble at least for the unfolded species (Figure 1). Already in 1959, the Zimm–Bragg theory described the helix–coil transition of polypeptides as the successive, dependent breakdown of ordered segments into disordered ones, rather than a simple two-state “all-or-nothing” mechanism.^1^ According to Malhotra & Udgaonkar,^2^ the folding of several proteins is multisteps with stable folding intermediates that accumulate and become easily detectable, but most of the papers the authors quote are theoretical hypotheses or tentative experimental approaches.^3–15^ Also in early unfolding studies, many researchers hypothesized the existence of one or more intermediate, albeit usually invisible, conformers, their invisibility being linked mainly to their low population.^16–19^ During the unfolding process, there are often minor transitions involving local regions of the secondary structure outside the hydrophobic core. Last but not least, it is important to recall that there is never a single unique unfolded state, which is an ensemble of different conformations.

**Figure 1:**
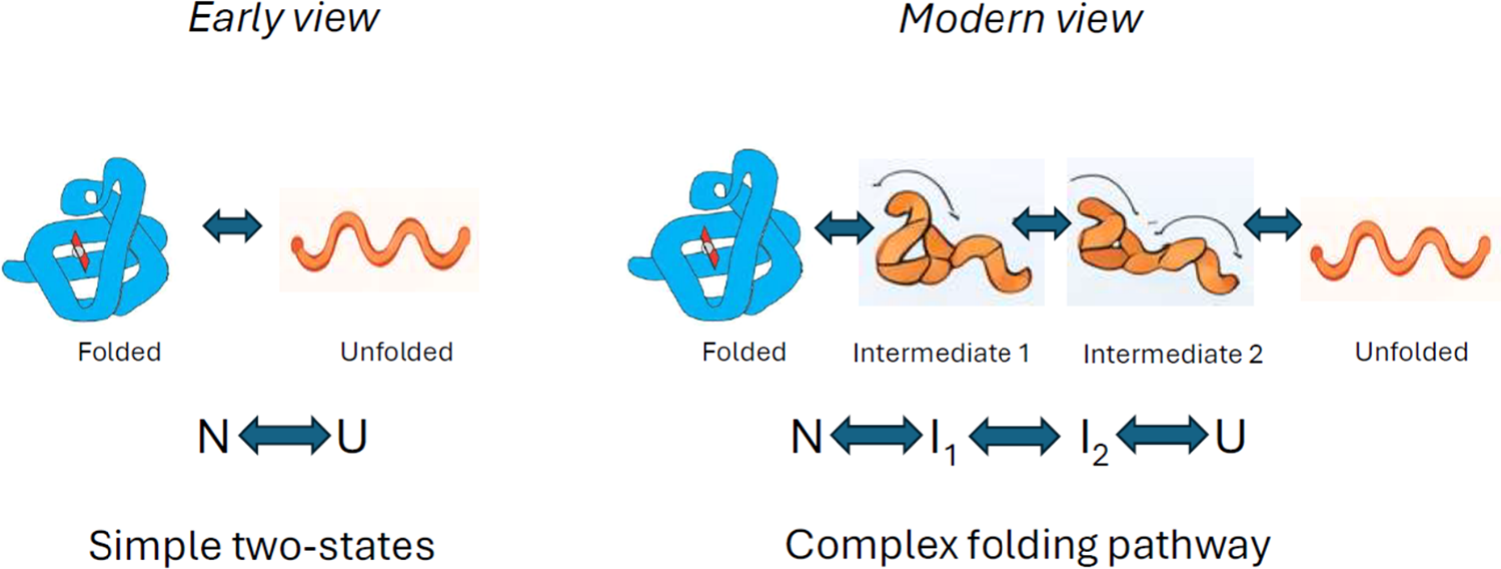
Cartoon illustrating the difference between the two-state model and contemporary view of unfolding.

In the present Mini-Review, we shall examine all of these aspects of this fascinating and important problem. We aim to discuss recent developments framed within a historical perspective. Together, the large plethora of new data motivates a conceptual shift: protein unfolding should be viewed not as a single, well-defined transition but as a family of related processes that expose different facets of conformational plasticity. Understanding this complexity is essential for connecting unfolding to folding, misfolding, aggregation, and functional conformational change and for developing a unified, physically grounded description of protein energy landscapes.

## Visible Conformations

The importance of intermediate conformations during protein folding/unfolding has been discussed in an endless number of experimental studies. As recently recalled, in 1983, Ohgushi and Wada described a partially folded compact intermediate with native-like secondary structure but a dynamic, flexible tertiary structure and named it “molten globule.”^20^ This concept inspired generations of scientists to identify and describe the structures of these intermediates. The opposite concept, that of an (unfolding) intermediate with defined hydrophobic core but no secondary structure, was explored instead in a well-known example by Zaidi et al.^21^ who identified two distinct intermediate states during the chemical unfolding of barstar. The authors described an intermediate with a secondary structure similar to the folded form but with a partially solvated hydrophobic core, whereas the other had no detectable secondary structure but conserved a hydrophobic core. Their findings suggested that the unfolding pathway of this protein does not follow a simple two-state model between the native and fully unfolded forms, but rather proceeds through multiple partially structured intermediates.

However, interpreting intermediates detected under chemical denaturation conditions requires caution. The “aggressive” nature of chemical denaturants, e.g., guanidinium chloride or urea, greatly increase the likelihood of trapping or “freezing” transient, non-native, and possibly irrelevant conformations. As a result, it becomes challenging to determine whether the observed intermediates truly correspond to states that lie on the natural unfolding pathway of the protein, or whether they are artifacts produced by the denaturing conditions themselves.

Single-molecule and mass-spectrometric approaches now directly detect low-population intermediates under both native and denaturing conditions, revealing species invisible to ensemble probes.^22^ For example, urea-induced unfolding monitored by high-resolution MS captures transient intermediates that reflect stepwise structural loss rather than cooperative collapse.

In general, any unfolding originating from chemical agents can produce results that are different from typical unfolding. For instance, Sahin et al.^23^ found that globular proteins, under alkali conditions, are destabilized rather than globally unfolded as revealed by native ion mobility-mass spectrometry. Structural work has resolved well-defined intermediate conformations, supporting their mechanistic relevance on the unfolding pathway.^24^ However, condition-dependent intermediates remain as a significant interpretive challenge. Chemical denaturants can reshape the energy landscape and stabilize conformers not populated during physiological unfolding; indeed, some intermediates appear only under urea or guanidinium but not during thermal unfolding. These observations underscore the need to distinguish genuine on-pathway intermediates from denaturant-stabilized artifacts and highlight the value of methods that probe unfolding without strongly perturbing the native landscape.

## Invisible Conformations

A new field related to intermediate conformations was developed by Lewis Kay in collaboration with the Dobson’s group.^25^ The authors tried to identify an intermediate conformation of Fyn SH3, the SH3 domain from Fyn tyrosine kinase, using relaxation NMR spectroscopy.

At variance with the description of the unfolding process as according to a two-state model, Korzhnev et al.^25^ were able to describe an intermediate conformation with a population of ca. 1% with respect to all molecules. The same authors described the detailed structure of this intermediate. The structure of the invisible species looks very similar to that of the experimental structure of folded Fyn SH3. A few years later, further studies on intermediate conformations, based on relaxation NMR spectroscopy and finalized to describe the folding funnel, were summarized by Zhuravleva and Korzhnev in a review.^26^

The laboratory of L.E. Kay, after the first paper in 2004, published several other papers based on CEST.^27–31^ De et al.^32^ described for instance an intermediate funnel for the four-helix bundle FF domain (71 residues) from the human protein HYPA/FBP11. Using chemical exchange saturation transfer (CEST) NMR experiments, the authors presented an atomic-resolution structure of a transient intermediate (dubbed I2) that along with the structure of a previously identified intermediate (dubbed I1) provided information on the whole FF domain folding trajectory. From the same laboratory, Tiwari et al.^27^ expanded the analysis of the FF domain from HYPA/FBP11 to obtain a further characterization of the exchange of a visible conformer with two invisible states. Tiwari et al.^27^ showed that a three-state exchange model could not be obtained by analysis of data recorded at a single temperature, but a simple two-state analysis of CEST data recorded at multiple temperatures resulted in a robust model of a three-state exchange between the F, I1, and I2 states interconverting in the millisecond time scale.

Always using chemical exchange saturation transfer (CEST) methods, Tiwari and co-workers^33^ investigated the L99A cavity variant of T4 lysozyme. Previous Carr–Purcell–Meiboom–Gill (CPMG) relaxation dispersion measurements had established that this protein undergoes millisecond time scale exchange between two compact conformations: a predominant native form, in which the side chain of Phe114 is solvent-exposed (E state), and a low-population alternative, in which the same aromatic group is sequestered within the protein interior (B state, ∼2%). Molecular dynamics simulations capable of reproducing the transition between these conformations have suggested the presence of additional compact states beyond E and B, although such species had not been observed despite extensive CPMG analyses. By examining ^15^N amide and ^13^CH_3_ CEST profiles for L99A T4L, the authors identified not only the known E and B states but also an additional, sparsely populated intermediate (I), present at roughly 0.2% at 11.5 °C, which exchanges rapidly with the B state.

In a subsequent study, Khandave et al.^34^ emphasized that successful detection of low-population states by CEST is strongly influenced by the selection of appropriate saturation field strengths (B_1_) but also depends critically on relaxation properties. In particular, they demonstrated that the transverse relaxation rate of the minor species (R_2_,_B_) significantly affects which B_1_ values yield the most informative results. Their analysis highlighted the importance of accounting for relaxation effects when designing CEST experiments, especially in cases involving slow exchange processes, sites with inherently high relaxation rates, or situations where minor state features are broadened due to exchange among multiple low-population conformers.

## External Transitions

Most experimental methods used to study protein unfolding lack spatial resolution and provide instead a global measure of stability. These approaches typically track changes in overall structural features, as for instance secondary structure elements, in response to environmental perturbations. For example, CD spectroscopy detects unfolding through changes in signal intensity associated with the loss of α-helices and β-sheets under environmental perturbations. However, such techniques do not reveal how individual regions of a protein respond during unfolding. A more detailed understanding can be achieved by examining residue-specific behavior. Two-dimensional NMR spectroscopy is, for instance, particularly suited for this purpose, as it allows monitoring of resonances arising from individual residues and a large number of studies have made use of this approach (Figure 2). Variations in peak volumes can be directly linked to local structural changes, providing insight into how specific residues are affected throughout the unfolding process.^35^ In a recent study, Puglisi et al. investigated the unfolding of a small yeast protein that undergoes both cold and heat denaturation at detectable temperatures at the level of individual residues across the entire protein, including those located outside the core.^36^ This broader analysis revealed that not all residues behave uniformly: some follow the expected cooperative two-state transition, while others display more complex thermodynamic profiles. As a result, residues can be divided into two groups: those consistent with an average unfolding behavior and those that deviate significantly. The two sets tell of course a slightly different story of how the unfolding process takes place.

**Figure 2:**
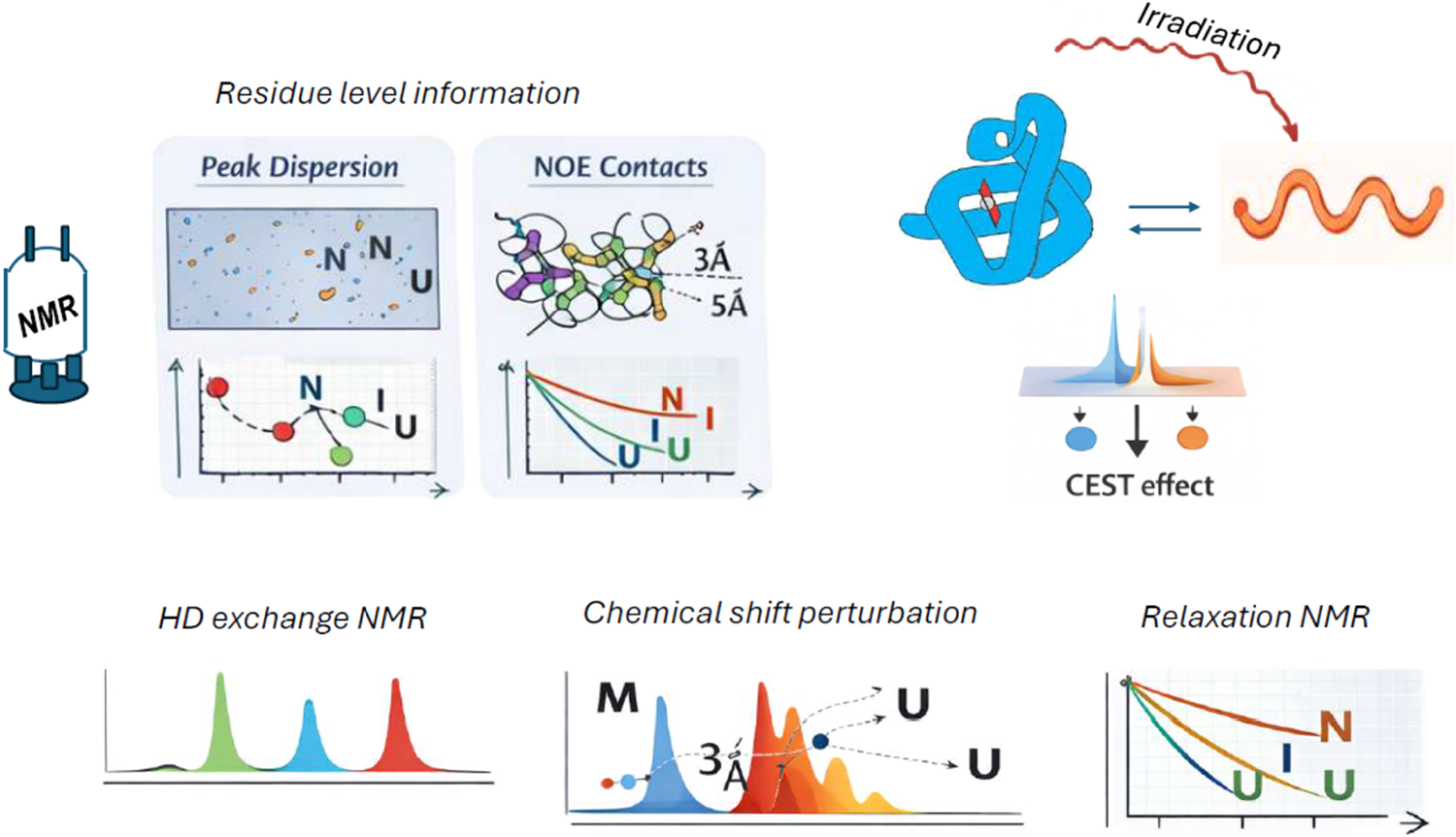
Cartoon illustration of NMR experiments obtainable from NMR spectroscopy.

A key finding of this study was the distinct difference between heat- and cold-induced unfolding. Residues that deviated from the average stability curve tended to exhibit increased stability at low temperatures and only modest destabilization at high temperatures. This asymmetry supported the model proposed by Privalov,^37^ which attributes cold denaturation to the hydration of hydrophobic residues within the protein core. According to this model, water penetration disrupts hydrophobic interactions, leading to destabilization at low temperatures, whereas heat denaturation is primarily driven by an increased conformational entropy. An additional observation of the Puglisi et al. study was that some solvent-exposed residues remain structurally intact under conditions where core residues appeared to have already unfolded. This suggested that unfolding at a low temperature is not uniform across the protein. Instead, the hydrophobic core may destabilize first due to increased hydration, while more-exposed regions lose structure at later stages. Together, these findings highlight the complex and heterogeneous nature of protein unfolding and demonstrate the value of site-specific techniques in uncovering details that are not accessible through global measurements.

## Relationship between Temperature and Pressure Unfolding

The relationship between different types of unfolding is an important topic that has been extensively covered by several authors, Meersman et al. (2002) had, for instance, thoroughly discussed the importance of a thermodynamic theory describing the pressure versus temperature diagram (i.e., the phase diagram) to explain the heat, cold, and pressure denaturation transitions in a coherent picture, creating an important pillar for these studies.^38^ Major advancements in the field were achieved over the last 20 years by combining small-angle X-ray scattering and nuclear NMR techniques. Both approaches allow the possibility of increasing pressure in the relative cells and reaching high values up to several thousand atmospheres. Which are the values usually needed to unfold small, compact globular proteins? Extensive studies by relatively few groups have been carried out using high-pressure nuclear magnetic resonance and have emphasized evidence that some very stable states can only be reached through pressure application.^39,40^ It is in fact clear that there is a synergy between pressure- and temperature-induced denaturation: increasing the pressure can, for instance, allow one to elevate the cold denaturation transition moving it to detectable temperatures near or above the water freezing point. In a recent study appearing in 2025,^41^ Roumestand et al. compared the cold, heat, and pressure-unfolded states and demonstrated what had up to then been only a hypothesis: the pressure-unfolded NMR spectrum recorded at room temperature shares features in common with that of the low but not the high temperature and room pressure state, suggesting a tighter similarity of the mechanisms in these different processes. This study has thus helped to advance our understanding of the mechanisms that induce different processes of unfolding under different conditions.

## Future Outlook

We have critically discussed the current state of our perception not only of the protein folding state(s) but also of the very process of unfolding which is a complex event that reveals much about protein structure and stability. It is clear that the historical assumption of a two-state unfolding is completely unrealistic at the light of the experimental evidence accumulated over several decades. However, despite the large plethora of experimental data already accumulated, protein unfolding studies remain important especially in the artificial intelligence (AI) era. As George Rose and co-workers elegantly put it in a recent review: “··· deep-learning AI represents a major advance in protein *fold* prediction. But this is not *folding* prediction. Patterns extracted from proteins in the Protein Data Bank (PDB) provide a ready “parts list,” circumventing the folding process entirely. [···] The situation is analogous to interpreting a movie by fast-forwarding to the final scene without first watching the previous 2 h; we know how it ends, but we do not know why.”^42^ Although, here, we have been discussing not folding but unfolding, it is equally true that we need to watch the whole movie to fully understand the process and recognize the different stages that it implies. This is the goal that thus awaits us in the near future. It is only in this way that we will be able to understand the forces that govern protein folding/unfolding and find ways to circumvent misfolding.

## References

[1] Zimm B. H., Bragg J. K. (1959). Theory of the Phase
Transition between Helix and Random
Coil in Polypeptide Chains. J. Chem. Phys..

[2] Malhotra P., Udgaonkar J. B. (2016). How cooperative
are protein folding and unfolding transitions?. Protein Sci..

[3] Khorasanizadeh S., Peters I. D., Roder H. (1996). Evidence for a three-state model
of protein folding from kinetic analysis of ubiquitin variants with
altered core residues. Nat. Struct Mol. Biol..

[4] Pradeep L., Udgaonkar J. B. (2002). Differential
salt-induced stabilization of structure
in the initial folding intermediate ensemble of Barstar. J. Mol. Biol..

[5] Poland D. C., Scheraga H. A. (1965). Statistical mechanics of noncovalent
bonds in polyamino
acids. IX. The two-state theory of protein denaturation. Biopolymers.

[6] Kim P. S., Baldwin R. L. (1982). Specific intermediates in the folding
reactions of
small proteins and the mechanism of protein folding. Annu. Rev. Biochem..

[7] Roder H., Elove G. A., Englander S. W. (1988). Structural
characterization of folding
intermediates in cytochrome c by H-exchange labelling and proton NMR. Nature.

[8] Kim P. S., Baldwin R. L. (1990). Intermediates in the folding reactions of small proteins. Annu. Rev. Biochem..

[9] Zitzewitz J. A., Matthews C. R. (1993). Protein engineering
strategies in examining protein
folding intermediates. Curr. Opin Struct Biol..

[10] Georgescu R. E., Li J.-H., Goldberg M. E., Tasayco M. L., Chaffotte A. F. (1998). Proline
isomerization-independent accumulation of an early intermediate and
heterogeneity of the folding pathways of a mixed α/β protein, *Escherichia coli* thioredoxin. Biochemistry.

[11] Brockwell D. J., Radford S. E. (2007). Intermediates:
ubiquitous species on folding energy
landscapes?. Curr. Opin. Struct. Biol..

[12] Patra A. K., Udgaonkar J. B. (2007). Characterization
of the folding and unfolding reactions
of single-chain monellin: evidence for multiple intermediates and
competing pathways. Biochemistry.

[13] Orte A., Craggs T. D., White S. S., Jackson S. E., Klenerman D. (2008). Evidence of
an intermediate and parallel pathways in protein unfolding from single-molecule
fluorescence. J. Am. Chem. Soc..

[14] Pirchi M., Ziv G., Riven I., Cohen S. S., Zohar N., Barak Y., Haran G. (2011). Single-molecule
fluorescence spectroscopy maps the folding landscape
of a large protein. Nat. Commun..

[15] Otosu T., Ishii K., Tahara T. (2015). Microsecond protein dynamics observed
at the single-molecule level. Nat. Commun..

[16] Camacho C. J., Thirumalai D. (1993). Kinetics and
thermodynamics of folding in model proteins. Proc. Natl. Acad. Sci. U.S.A..

[17] Dill K. A., Fiebig K. M., Chan H. S. (1993). Cooperativity
in protein-folding
kinetics. Proc. Natl. Acad. Sci. U.S.A..

[18] Sali A., Shakhnovich E., Karplus M. (1994). How does a protein fold?. Nature.

[19] Onuchic J. N., Wolynes P. G., Luthey-Schulten Z., Socci N. D. (1995). Toward an outline
of the topography of a realistic protein-folding funnel. Proc. Natl. Acad. Sci. U.S.A..

[20] Ohgushi M., Wada A. (1983). Molten-globule state’:
a compact form of globular proteins
with mobile side-chains. FEBS Lett..

[21] Zaidi F. N., Nath U., Udgaonkar J. B. (1997). Multiple
intermediates and transition
states during protein unfolding. Nat. Struct.
Biol..

[22] Österlund N., Jordan J. S., Renzi E., Szekeres G. P., Pagel K. (2025). Mass spectrometry
detects folding intermediates populated during urea-induced protein
denaturation. Chem. Sci..

[23] Sahin C., Österlund N., Leppert A., Johansson J., Marklund E. G., Benesch J. L. P., Ilag L. L., Allison T. M., Landreh M. (2021). Ion mobility-mass spectrometry shows stepwise protein
unfolding under alkaline conditions. Chem. Commun.

[24] Masoumzadeh E., Courtney J. M., Charlier C., Ying J., Anfinrud P., Bax A. (2025). Structure
of a transient protein-folding intermediate by pressure-jump
NMR spectroscopy. Proc. Natl. Acad. Sci. U.S.A..

[25] Korzhnev D. M., Salvatella X., Vendruscolo M., Di Nardo A. A., Davidson A. R., Dobson C. M., Kay L. E. (2004). Low-populated folding intermediates
of Fyn SH3 characterized by relaxation dispersion NMR. Nature.

[26] Zhuravleva A., Korzhnev D. M. (2017). Protein folding
by NMR. Prog.
Nucl. Magn. Reson. Spectrosc..

[27] Tiwari V. P., De D., Thapliyal N., Kay L. E., Vallurupalli P. (2024). Beyond slow
two-state protein conformational exchange using CEST: applications
to three-state protein interconversion on the millisecond timescale. J. Biomol. NMR.

[28] Bolik-Coulon N., Hansen D. F., Kay L. E. (2022). Optimizing
frequency sampling in
CEST experiments. J. Biomol. NMR.

[29] Karunanithy G., Yuwen T., Kay L. E., Hansen D. F. (2022). Towards autonomous
analysis of chemical exchange saturation transfer experiments using
deep neural networks. J. Biomol. NMR.

[30] Tiwari V. P., Toyama Y., De D., Kay L. E., Vallurupalli P. (2021). The A39G FF
domain folds on a volcano-shaped free energy surface via separate
pathways. Proc. Natl. Acad. Sci. U.S.A..

[31] Yuwen T., Bouvignies G., Kay L. E. (2018). Exploring methods to expedite the
recording of CEST datasets using selective pulse excitation. J. Magn. Reson..

[32] De D., Thapliyal N., Tiwari V. P., Toyama Y., Hansen D. F., Kay L. E., Vallurupalli P. (2024). Mapping the FF domain folding pathway
via structures of transiently populated folding intermediates. Proc. Natl. Acad. Sci. U.S.A..

[33] Tiwari V. P., Khandave N. P., Hansen D. F., Bouvignies G., Kay L. E., Vallurupalli P. (2026). Using CEST NMR to Discover Previously
Unobserved States on the Free Energy Surface of Proteins: Application
to the L99A Cavity Mutant of T4 Lysozyme. J.
Biol. Chem..

[34] Khandave N. P., Hansen D. F., Vallurupalli P. (2024). Increasing the accuracy of exchange
parameters reporting on slow dynamics by performing CEST experiments
with ’high’ B 1 fields. J. Magn.
Reson..

[35] Puglisi R., Karunanithy G., Hansen D. F., Pastore A., Temussi P. A. (2021). The anatomy
of unfolding of Yfh1 is revealed by site-specific fold stability analysis
measured by 2D NMR spectroscopy. Commun. Chem..

[36] Puglisi R., Brylski O., Alfano C., Martin S. R., Pastore A., Temussi P. A. (2020). Quantifying the
thermodynamics of protein unfolding
using 2D NMR spectroscopy. Commun. Chem..

[37] Privalov P. L. (1990). Cold denaturation
of protein. Crit. Rev. Biochem. Mol. Biol..

[38] Meersman F., Smeller L., Heremans K. (2002). Comparative
Fourier transform infrared
spectroscopy study of cold-, pressure-, and heat-induced unfolding
and aggregation of myoglobin. Biophys. J..

[39] Rouget J. B., Schroer M. A., Jeworrek C., Pühse M., Saldana J. L., Bessin Y., Tolan M., Barrick D., Winter R., Royer C. A. (2010). Unique features of the folding landscape
of a repeat protein revealed by pressure perturbation. Biophys. J..

[40] Knop J. M., Mukherjee S., Jaworek M. W., Kriegler S., Manisegaran M., Fetahaj Z., Ostermeier L., Oliva R., Gault S., Cockell C. S., Winter R. (2023). Life in Multi-Extreme Environments:
Brines, Osmotic and Hydrostatic Pressure–A Physicochemical
View. Chem. Rev..

[41] Roumestand C., Dudas E., Puglisi R., Calió A., Barthe P., Temussi P. A., Pastore A. (2025). Understanding
the Relationship
between Pressure and Temperature Unfolding of Proteins. JACS Au.

[42] Chen S. J., Hassan M., Jernigan R. L., Jia K., Kihara D., Kloczkowski A., Kotelnikov S., Kozakov D., Liang J., Liwo A., Matysiak S., Meller J., Micheletti C., Mitchell J. C., Mondal S., Nussinov R., Okazaki K. I., Padhorny D., Skolnick J., Sosnick T. S., Stan G., Vakser I., Zou X., Rose G. D. (2023). Opinion: Protein
folds vs. protein folding: Differing questions, different challenges. Proc. Natl. Acad. Sci. U.S.A..

